# Advanced Bioresin Formulation for 3D-Printed Bone Scaffolds: PCLDMA and p-PLA Integration

**DOI:** 10.3390/polym16040534

**Published:** 2024-02-16

**Authors:** Deniz Sakarya, Tolga Zorlu, Sevil Yücel, Yesim Muge Sahin, Ali Can Özarslan

**Affiliations:** 1 Institute of Nanotechnology and Biotechnology, İstanbul University-Cerrahpaşa, Istanbul 34500, Turkey; 2 Faculty of Chemistry-Metallurgy, Bioengineering Department, Yildiz Technical University, Istanbul 34210, Turkey; s.yucel@yildiz.edu.tr (S.Y.); alicanozarslan@gmail.com (A.C.Ö.); 3 Faculty of Chemistry, Institute of Functional Materials and Catalysis, University of Vienna, 1090 Vienna, Austria; tolga.zorlu@univie.ac.at; 4 Polymer Technologies and Composite Application and Research Center (ArelPOTKAM), Istanbul Arel University, Buyukcekmece, Istanbul 34537, Turkey; ymugesahin@arel.edu.tr

**Keywords:** bone tissue scaffold, bone regeneration, composite materials, polycaprolactone dimethacrylate (PCLDMA), polylactic acid (PLA)

## Abstract

In bone tissue engineering, scaffold attributes such as pore dimensions and mechanical strength are crucial. This study synthesized polycaprolactone dimethacrylate (PCLDMA) from polycaprolactone (PCL), incorporating epichlorohydrin (Epi-PCL) and methacryloyl chloride (Meth-Cl). PCLDMA was blended with polylactic acid (p-PLA) to 3D-print bone scaffolds using stereolithography (SLA). Analytical techniques included nuclear magnetic resonance (NMR), Fourier-transform infrared spectroscopy (FTIR), scanning electron microscopy (SEM), and compression testing. Degradation kinetics and cell viability were investigated using human osteoblast (HOB) cells. Findings revealed PCLDMA/p-PLA composite scaffold superiority over the original polymers. Notably, PCLDMA-60 (60% PCLDMA, 40% p-PLA) displayed optimal properties. Compressive strength varied from 0.019 to 16.185 MPa, porosity from 2% to 50%, and degradation rates from 0% to 0.4% over three days. Cell viability assays affirmed biocompatibility across various PCLDMA ratios. In conclusion, PCLDMA/p-PLA composite scaffolds, particularly PCLDMA-60, show great potential in bone tissue engineering.

## 1. Introduction

Bone ranks as the second most frequently transplanted tissue, following hematopoietic stem cell transplantation [1]. Defects in bone tissue can be attributed to a range of factors, including traumatic injury, neoplastic growth, infection, and others, frequently necessitating clinical intervention. The regenerative processes associated with bone healing exhibit a comparatively protracted temporal course when compared to the repair mechanisms operative in other tissue types. These inherent characteristics augment the susceptibility to complications during therapeutic interventions. Prolonged or incomplete recuperation carries the potential for the emergence of acute or persistent physiological and functional impairments in the afflicted patient, thereby exerting a pronounced influence on the patient’s overall quality of life. One of the principal modalities employed in the restitution and fortification of impaired osseous structures entails the deployment of bone grafts. This therapeutic approach enjoys widespread utilization within the domain of orthopedics, with applications encompassing fracture management as well as the restitution of skeletal losses precipitated by infectious etiologies or tumorigenic processes. These concerns notwithstanding, in light of the manifold shortcomings associated with conventional treatment strategies such as autografts, allografts, and xenografts, scholars have increasingly redirected their investigative endeavors toward the realm of synthetic bone graft materials [2].

The primary factor to consider while acquiring synthetic bone transplant materials is the careful selection of the components. The selected material must accurately mimic the structural and functional characteristics of natural bone tissue, while also meeting essential requirements such as biocompatibility, non-toxicity, the ability to promote continuous bone tissue regeneration, and the display of mechanical properties equivalent to those of native bone tissue. Polymers made from polycaprolactone (PCL), as well as materials based on PCL, have shown impressive effectiveness in imitating the characteristics of bone tissue. Due to its high mechanical strength, biocompatibility, slow degradation rate, and easy ability to be shaped, PCL is considered the top choice among polymers for making synthetic bone grafts [3,4,5]. Polylactic acid (PLA) is another polymer that is highly favored in the field of biological applications. In addition to its impressive biocompatibility, non-toxicity, and appropriate mechanical strength [6,7], PLA stands out due to its somewhat slower degradation rate compared to PCL polymers [8,9,10]. This feature allows for deliberate degradation by adjusting the proportions of the components. The combination of these characteristics makes PLA a highly advantageous addition to PCL polymers in creating composite polymer systems specifically designed for producing synthetic bone graft materials [11,12,13,14].

Selecting the most effective production method is a crucial decision in the field of tissue engineering. Traditional techniques, such as gas foaming, solvent casting, electrospinning, phase separation, freeze casting, freeze drying, and others, have traditionally been used to create consistent individual scaffolds. Nevertheless, these traditional methods have certain constraints when it comes to the precise control of important factors, particularly including geometric shape, pore size, porosity, and interstitial connectivity [15,16,17,18]. Regenerating damaged regions with specific biological and physicochemical requirements, such as subchondral bone and cartilage, presents numerous problems [19].

The development of additive manufacturing technology, also known as 3D printing, has been designed to address the limitations outlined above. This technology enables the creation of customized scaffolds that are designed to meet specific anatomical requirements. It also allows for the fabrication of multi-layered matrices that have a variety of microstructural compositions and material components. In addition, 3D printing offers various benefits, such as the ability to print directly within the affected area, increased accuracy, faster creation of support structures, and a resulting decrease in production costs [20,21,22]. Layered-manufactured scaffolds mimic the properties of natural extracellular matrices (ECM), creating an ideal environment for cell growth, efficient nutrition transport, effective waste removal, and the spread of growth hormones. In addition, they facilitate the development of internal vascular networks [23,24]. The utilization of 3D printing technology provides a compelling opportunity to produce synthetic matrices with complex geometries, which are highly compatible with stem cells and growth factors. This accelerates the process of tissue regeneration. Choosing the best 3D printing technology for making bone scaffolds depends on factors including speed of production, ability to create detailed structures, and the characteristics of the materials used. The main cell-free 3D printing methods utilized in medical applications are selective laser sintering (SLS), stereolithography (SLA), and fused deposition modeling (FDM) [17]. These approaches have shown significant progress, allowing for the accurate creation of structures using computer-aided designs and medical imaging data. Stereolithography stands out for its versatility in creating structures of various sizes, ranging from centimeters to submicron dimensions. It provides significantly higher accuracy and detail compared to other solid freeform fabrication (SFF) methods. While most conventional fabrication techniques produce features that are typically between 50–200 µm in size, several commercially available stereolithography systems have shown the ability to create objects that are several cubic centimeters in size with a remarkable accuracy of 20 µm. These breakthroughs have enabled the creation of models tailored to individual patients, complex surgical parts, and customized items such as hearing aids and implants supported by molds, all within the field of biomedicine.

While extensive research has been conducted within the realm of this topic [25,26,27], there is a conspicuous gap in the existing literature pertaining to the endeavor to enhance the ultraviolet (UV) curability of the PCL polymer through modification and its subsequent application. This particular study involved the synthesis of a photocurable polymer denoted as polycaprolactone dimethacrylate (PCLDMA), derived from an 80,000 molecular weight PCL polymer, via the incorporation of epichlorohydrin and methacryloyl chloride. By combining epoxidized soybean oil (ESO) and acrylic acid, acrylated epoxidized soybean oil (AESO) was produced for use as a crosslinker. Subsequently, the synthesized PCLDMA polymer was blended with a commercially available photocurable resin, named p-PLA, and with AESO as a crosslinker. Diphenyl(2,4,6-trimethylbenzoyl) phosphine oxide (TPO) was incorporated as a photoinitiator. The proportions of p-PLA were varied to formulate a bioresin intended for the production of bone scaffolds. Different compositions of scaffolds were achieved through the utilization of a stereolithography (SLA) 3D printer. Comprehensive structural analyses, encompassing nuclear magnetic resonance (NMR) and Fourier-transform infrared spectroscopy (FTIR), were conducted. Morphological evaluations were carried out employing scanning electron microscopy (SEM), while mechanical attributes were assessed via compression testing. Furthermore, investigations involving degradation kinetics were performed, as well as assessments pertaining to cell viability and adhesion, by utilizing the human osteoblast (HOB) cell line on the resultant bone scaffolds.

## 2. Experimental Section

A commercial photopolymer resin composed of polylactic acid (PLA), denoted as p-PLA, was procured from Shenzhen Esun Industrial Co., Ltd. (Shenzhen, China) Epoxidized soybean oil (ESO) was obtained from Akdeniz Chemson Chemical Industry and Trade Inc. (Izmir, Turkey). Unless explicitly stated otherwise, all additional chemicals and solvents were acquired from Sigma-Aldrich.

### 2.1. Synthesis of Methacrylated PCL

**Figure 1:**
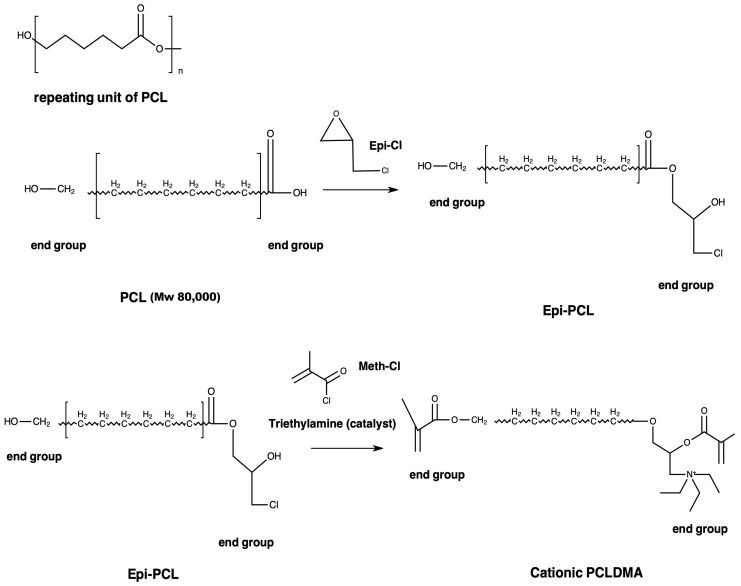
Synthesis procedure of PCLDMA.

PCL (Mn: 80,000 g/mol) was reacted with epichlorohydrin (Epi-Cl) to increase the number of -OH groups in the polymer chain. Briefly, PCL was dissolved in toluene at a ratio of 2:5 (*w*/*v*) at 90 °C and 1 mL Epi-Cl was added to mixture. After 4 h, toluene was evaporated. The resulting macromer (Epi-PCL) was dissolved in tetrahyrofurane (THF) at a ratio of 1:10 (*w*/*v*). In the presence of an equal molar amount of triethylamine (TEA), methacryloyl chloride (Meth-Cl) was added to the mixture to neutralize methacrylic acid and photocurable polycaprolactone-dimethacrylate (PCLDMA) was produced (Figure 1). TEA was used to neutralize the methacrylic acid formed in the reaction. Methacrylation reaction was carried at room temperature. After the completion of the reaction, THF, TEA, and the unreacted Meth-Cl and methacrylic acid formed in the reaction were cast out by precipitating the macromer in dichloromethane (DCM). The isolated macromer was washed 3 times with ultra-pure water and vacuum-dried overnight. The macromer was named polycaprolactone dimethacrylate (PCLDMA).

### 2.2. Synthesis of Acrylated Epoxidized Soybean Oil (AESO)

**Figure 2:**
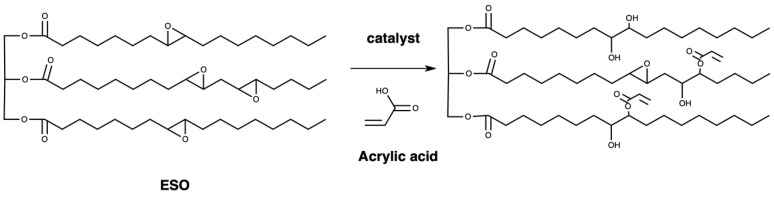
Formation of epoxidized soybean oil-acrylic acid ester.

In total, 100 g of ESO and 0.1 g of hydroquinone (purity ≥ 99.5) were joined to a flask with reflux condenser, a mechanical stirrer, and thermometer. The heat of mixture was arranged to 100 °C in an oil bath. Acrylic acid (22.7 g) was dripped into the flask. After adding the acrylic acid, stirring continued for 6 h until the reaction stopped. The utilization of the carboxyl group of acrylic acid and epoxy ring took place in the reaction [28]. The product, epoxidized soybean oil-acrylic acid ester (AESO), was obtained and used as a crosslinker for bioresins after cooling at room temperature. The experimental process is shown in Figure 2.

### 2.3. Fabrication Process of Scaffolds Using SLA

#### 2.3.1. Formulation of Bioresins

Eleven bioresin formulations were prepared by blending various ratios of PCLDMA, p-PLA, 40% (*w/w)* AESO, and 30% (*w/w)* photoinitiator Diphenyl(2,4,6-trimethylbenzoyl) phosphine oxide (TPO) in the dark at room temperature. PCLDMA/p-PLA ratios (*v*/*v*) for each sample are as shown in Table 1. To acquire resin with a suitable viscosity for use in the stereolithography process (similar to commercial resin viscosity, between 200–300 mPa.s), toluene was used.

#### 2.3.2. CAD Model of Scaffolds

**Figure 3:**
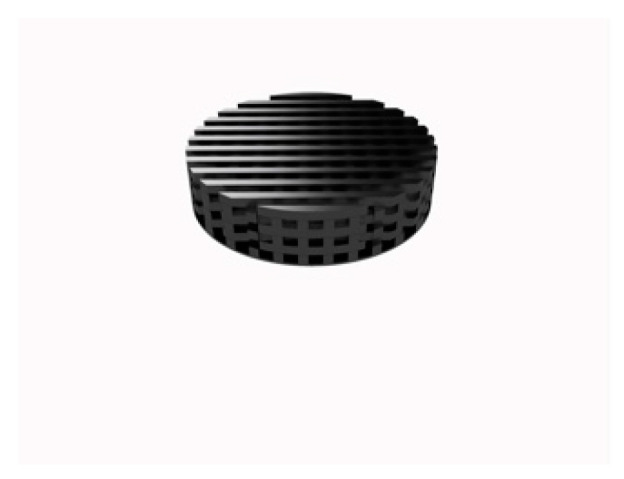
3D CAD design of bone tissue scaffolds.

A porous bone scaffold model was designed using computer-aided design (CAD) software (Autodesk Fusion 360, Autodesk Inc., San Rafael, CA, USA), as shown in Figure 3. The model was a cylinder with a radius of 10 mm and height of ~15 mm. The thickness of each layer designed at a 90° angle was 0.5 mm. The pores of the scaffold structure were arranged as 0.5 mm × 0.5 mm. The porosity of the scaffold models designed was 50%.

#### 2.3.3. 3D Printing of Scaffolds

Eleven resin formulations were printed using a Sonic Mini 4K Resin 3D Printer (Phrozen3DP, Taipei City, Taiwan). The parameters of the SLA 3D printer needed to be set before printing, such as the lifting speed (80 mm/min), bottom exposure time, slice thickness (0.05 mm), and exposure time. Bottom exposure time and exposure time for each sample were as follows, respectively: PCLDMA-100 (5 min and 5 min), PCLDMA-90 (55 s and 55 s), PCLDMA-80 (50 s and 50 s), PCLDMA-70 (45 s and 45 s), PCLDMA-60 (40 s and 40 s), PCLDMA-50 (30 s and 30 s), PCLDMA-40 (20 s and 20 s), PCLDMA-30 (13 s and 13 s), PCLDMA-20 (9 s and 9 s), PCLDMA-10 (5 s and 5 s), and PCLDMA-0 (3 s and 3 s). After the printing process finished, scaffolds were washed 3 times with an ethanol/distilled water mixture (1:1/*v*:*v*) to remove residual bioresin. Finally, the post-curing process was performed in an ultra-violet light box for 15 min.

### 2.4. Characterization of Macromers, Bioresins and Scaffolds

#### 2.4.1. Nuclear Magnetic Resonance (NMR) Spectroscopy

Nuclear magnetic resonance spectroscopy is a research technique primarily employed for structure determination, utilizing the specific magnetic properties of atomic nuclei. This technique facilitates the clear and precise differentiation of various functional groups within the analyzed structure. Additionally, it provides distinct signals that reveal the interactions between neighboring groups. Proton nuclear magnetic resonance spectroscopy (Varian UNITY INOVA 500 MHz, Varian Medical Systems, Inc.) was performed to characterize the Epi-PCL and PCLDMA. Polymer samples of 20 mg were solvated with 0.5 mL of deuterated chloroform and analyzed at 500 Hz at room temperature. Spectra peaks were referenced to CDCl_3_ at 7.26 ppm.

#### 2.4.2. Fourier-Transform Infrared (FTIR) Analysis PCLDMA and Photocurable PLA

Functional groups PCL, Epi-PCL, PCLDMA, and p-PLA were identified using a Fourier-transform infrared (FTIR) spectrometer. The FTIR analyses were conducted using a NICOLET 6700 FTIR spectrometer (Thermo Fisher Scientific, Waltham, MA, USA) equipped with an attenuated total reflectance (ATR) capability between 400 and 4000 cm^−1^ in wavenumber.

#### 2.4.3. Compressive Test of Scaffolds

Scaffolds underwent testing following the post-curing procedure. The compressive strength of the cylindrical in shape (20 mm × 15 mm) 3D composite scaffolds was measured at speed of 0.25 mm/min using an ALSA 100kN universal testing machine, equipped with a 2 kN load cell. Three specimens (*n* = 3) were tested for each experimental condition and data were presented as mean ± standard deviation (SD).

#### 2.4.4. In Vitro Degradation Test of Scaffolds

Three PCLDMA scaffolds, as they were printed, were utilized for the in vitro degradation study (*n* = 3). In cell-culture studies, PCL scaffolds would be pretreated between 3 and 24 h with sodium hydroxide (NaOH) to obtain more hydrophilic surface for cell attachment. For this purpose, bone tissue engineered 3D composite scaffolds were treated with 5*M* NaOH for 3 days.

The scaffolds were immersed in separate tubes with 10 mL of phosphate-buffered saline (PBS) (pH 7.4 at 37 °C at 5% CO_2_). At selected time points (2, 4, 8, 24, 48, and 72 h), the scaffolds were removed and rinsed with ultrapure water. The scaffolds were dried and placed in an oven at 37 °C for 12 h. The remaining mass was calculated according to Equation (1) [29]:

(1) $$Remaining\;mass\;\left(\%\right)\;=\;100\;-\;\frac{(M_{i}\;-\;M_{f})\;\times \;100}{M_{i}}$$

where *M_i_* is the initial mass of the sample and *M_f_* is the final mass of the sample after degradation.

#### 2.4.5. Surface Topography

Scanning electron microscopy (SEM) was used to observe the surface topography. For scanning electron microscopy (SEM), samples were coated with gold–palladium and examined in Thermo Fisher Quattro S at an accelerating voltage of 10–15 kV. The pore sizes of the fabricated scaffolds were measured from SEM images of 150×.

Porous materials find applications across various domains, spanning from engineering to medicine. Porosity and pore size significantly impact several characteristics of materials in these applications. For example, porosity plays a crucial role in fabricating three-dimensional (3D) scaffolds in tissue engineering. These scaffolds serve as templates for cells from various tissues in terms of how to attach, migrate, proliferate, and function. Therefore, it is essential to accurately quantify the porosity of these materials. Due to its significance, multiple methods have been developed to characterize porosity, including techniques based on Archimedes’ principle, BET (Brunauer–Emmett–Teller) analysis, and computerized tomographic (CT) imaging. Archimedes’ principle-based techniques and CT imaging methods are commonly employed for characterizing porous materials. The choice of technique depends on the physicochemical properties of the specific porous material under investigation. While micro-CT is widely accepted as a gold standard for porosity measurement, especially in biomedical applications, it may not be suitable for non-opaque materials. The limitations of BET surface area analysis include the potential for invalid equations due to the absence of a truly linear region, variations in sample size (especially in biomaterials and polymers), potential alterations in sample architecture caused by thermal preparation methods, limited gas adsorption in highly porous solid structures, and high associated costs. Calculations performed using the Image J program can also yield results with a high margin of error, especially when applied to materials with non-uniform surfaces or intricate details. Transitioning from area calculations to volume calculations in materials with variable heights may lead to inaccuracies. In contrast, Archimedes’ principle-based liquid displacement methods are highly straightforward, cost-effective, and feasible, making them a favorable choice [30]. An Archimedes’ principle-based liquid displacement method was used for the calculation of the porosity of scaffolds according to Equation (2):

(2) $$Porosity\;\left(\%\right)\;=\;100\;-\;\lfloor \frac{(V_{2}\;-\;V_{1})\;\times \;100}{V_{2}}\rfloor$$

where *V*_1_ is the volume change after immersing the sample with pores and *V*_2_ is the volume change after immersing the sample without pores.

### 2.5. Cell Culture

The human osteoblast (HOB) cell lines were obtained from the German Collection of Microorganisms and Cell Cultures (DSMZ) and maintained under sterile conditions. They were cultured in a medium enriched with 1% penicillin/streptomycin and 10% fetal bovine serum (FBS) in a 5% CO_2_ incubator at 37 °C.

#### 2.5.1. Cell Viability Assay

Cell viability tests were conducted in accordance with ISO 10993-5 guidelines, which outline test methods for assessing the in vitro cytotoxicity of medical devices. For this study, the HOB cell line was used instead of the L929 cell line typically employed in the standard test method. The extracts from the bone scaffolds, obtained using the SLA 3D printer, were prepared following the guidelines specified in ISO 10993-12. The assessment of resin cell viability was conducted through the MTT assay, which employs a water-soluble tetrazolium salt. The MTT molecule, upon entry into live cells, is exposed to enzymatic reduction by the mitochondrial enzyme succinate dehydrogenase, resulting in the formation of insoluble purple formazan crystals. The color intensity indicated is directly proportional to the number of living cells. HOB cell lines were grown in Dulbecco’s Modified Eagle’s medium (DMEM) augmented with 1.0% penicillin/streptomycin and 10% FBS. The in vitro toxic effects of bioresins were determined at 3 different concentrations (50, 70, and 100 μg/mL) over 72 h. Cells were seeded at a density of 4 × 10^3^ cells/well in 96-well tissue plates and allowed to reach 80% confluence over three days. Following this, cells were treated with different doses of bioresin extracts, prepared according to 10993-12, in a fresh medium and further incubated for the designated time periods. Cells were then exposed to a 10% MTT solution in the medium and incubated for 4 h. After incubation, the cells were treated with a 0.1 mg/mL sodium dodecyl sulphate (SDS) solution in 0.01 M HCl [31]. Absorbance readings were obtained at 570 nm using a Multiscan Go spectrophotometer (Thermo Fischer, Waltham, MA, USA)

#### 2.5.2. Fluorescent Microscopy

Cells were cultured with scaffold extracts and labelled with 4′,6-Diamidino-2-phenylindole dihydrochloride (DAPI) via a fluorescent microscope. Results were obtained and images were captured. Specifically, a chamber slide was seeded with 8 × 10^3^ cells and incubated for 48 h under standard cell culture conditions. Subsequently, the culture medium was replaced with a treatment medium containing DAPI. After an additional 15 min incubation, the cells underwent two washes with PBS, and images were acquired using a fluorescent microscope with DAPI filter illumination.

### 2.6. Statistical Analysis

Statistical evaluations were conducted utilizing one-way analysis of variance (ANOVA) accompanied by Tukey’s multiple comparison test for post hoc analysis. The research findings were presented in the form of mean values accompanied by their corresponding standard deviations (±SD). All experimental procedures were reiterated no fewer than three times. Significance levels were defined so that *p* values < 0.05 indicated statistical significance, while *p* values < 0.01 or 0.001 were indicative of a highly significant effect.

## 3. Results and Discussions

### 3.1. Synthesis and Properties of PCL Macromers

#### 3.1.1. PCL Macromer Characterization

**Figure 4:**
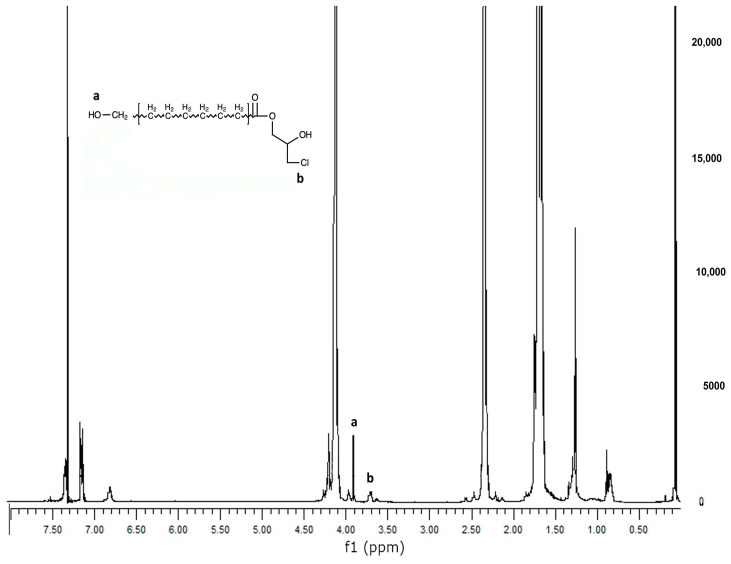
^1^H NMR spectrum of Epi-PCL.

**Figure 5:**
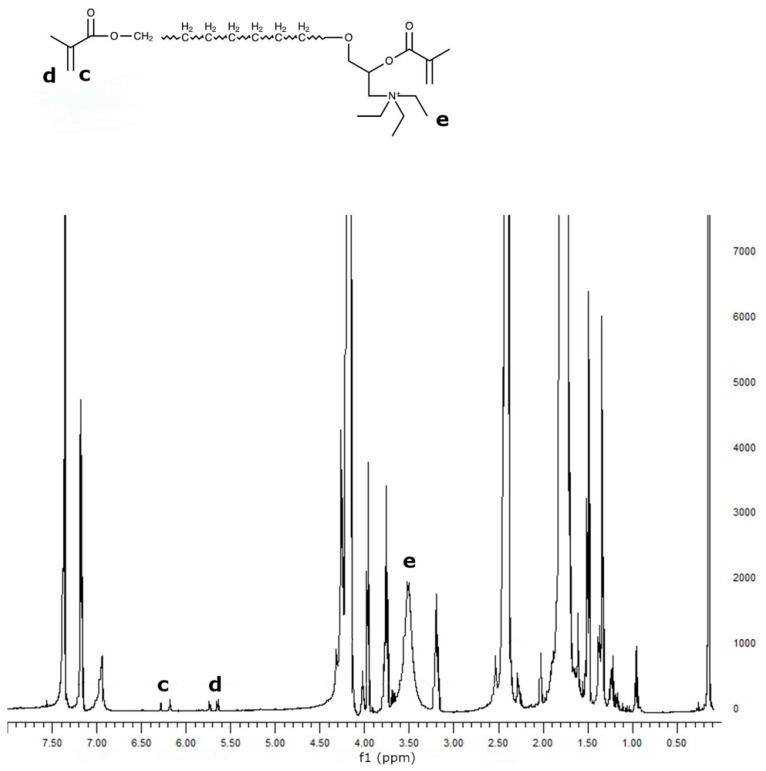
^1^H NMR spectrum of PCLDMA.

PCL was reacted with Epi-Cl to increase the number of -OH groups in the polymer chain, and then methacrylated via a reaction with Meth-Cl and triethylamine (TEA) to produce PCLDMA. Epi-PCL and PCLDMA macromer samples were analyzed via proton nuclear magnetic resonance (NMR) spectroscopy. Figure 4 and Figure 5 show spectra of Epi-PCL and PCLDMA respectively.

The characteristic peaks of PCL appeared at 1.7, 2.35, and 4.1 ppm. Hydrogens of α carbon to ester carbonyl appeared at 2.35 ppm. The hydrogens of the -O-CH_2_– group were revealed at 4.1 ppm [32]. When epichlorohydrin was heated with PCL, only free carboxyl groups of PCL would react with the oxirane ring of epichlorohydrin. The reaction product was a halogen containing ester. Hydrogens of -CH_2_-Cl groups were determined as having small peaks at 3.7 ppm. Additionally, the hydrogens of the free -OH group were found at 3.9 ppm. After methacrylation, methacrylate peaks were depicted at 5.6 and 6.1 ppm. The intensity of the peaks was very weak. When quaternarization was completed with triethylamine, new peaks appeared at 3.5 ppm. The results obtained are compatible with the literature and show that the synthesis has been carried out successfully [33].

#### 3.1.2. FTIR

**Figure 6:**
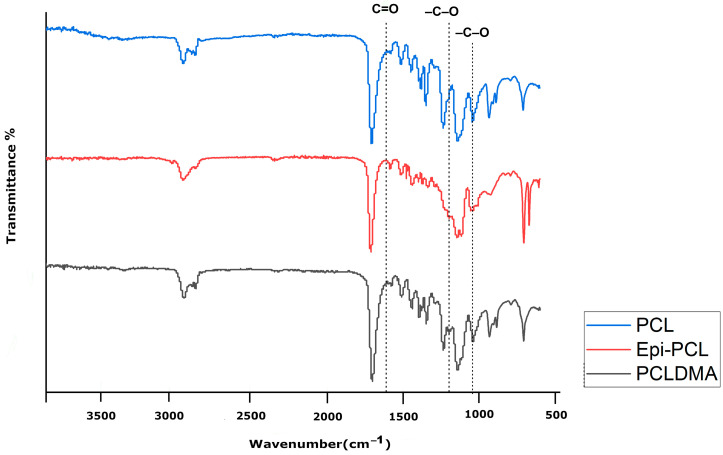
FTIR spectra of PCL, Epi-PCL, and PCLDMA.

Infrared spectroscopy is one of the most practical techniques used to modify macromolecules. Each functional group appears at a definite wavenumber in the FTIR spectra. Those numbers are generally specific and may change during modifications. FTIR spectra of PCL, Epi-PCL, and PCLDMA are shown in Figure 6. The characteristic peaks of PCL appear at 2948, 2873, 1721, 1258, 1163, 1065, 957, and 733 cm^−1^. The peaks at 2948 and 2873 indicated the presence of aliphatic -C-H groups. Carbonyl peaks of the ester group appeared at 1721 cm^−1^. The peaks that appeared at 1258, 1163, 1065, and 957 were related to the -C-O bending vibrations. The peak at 732 cm^−1^ was related to the rocking vibration of -CH_2_- groups [34]. When epichlorohydrin was introduced to PCL, the ester peak that appeared at 1721 cm^−1^ shifted to 1728 cm^−1^. Additionally, a peak appeared at 1065 cm^−1^ and new peaks appeared at 1075, 1043, and 1030 cm^−1^, probably due to the presence of -C-Cl bonds. After methacrylation, the value of the carbonyl peak changed to 1723 cm^−1^. The peak at 1220 cm^−1^ disappeared and the intensity of the 1065 cm^−1^ peak increased. A new peak was observed at 1140 cm^−1^ as a shoulder near the peak which appeared at 1163 cm^−1^. Also, the results support the NMR analysis.

### 3.2. Mechanical Properties of PCLDMA/p-PLA Composite Scaffolds

**Figure 7:**
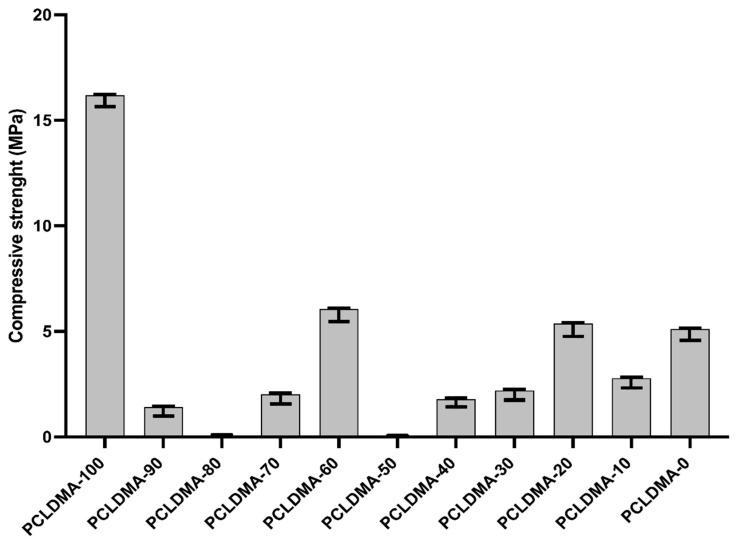
Compressive strength of PCLDMA/P-PLA composite 3D-printed scaffolds with various contents of PCLDMA (data are given as mean ± SD (*n* = 3)).

Previous research has suggested that alterations in the mechanical characteristics of the extracellular matrix (ECM) can modulate cellular behaviors, including cell spreading, morphology, and gene expression [35]. In Figure 7, the compressive strengths of PCLDMA/p-PLA composite 3D-printed scaffolds with varying PCLDMA contents were presented. Specifically, the compressive strength of these composite scaffolds ranged from 0.01 MPa to 16.18 MPa. In contrast, the compressive strength of a pure p-PLA 3D-printed scaffold was 5.11 MPa, compared to that of a pure PCLDMA 3D-printed scaffold at 16.18 MPa.

The sample named PCLDMA-60, consisting of 60% PCLDMA and 40% p-PLA, exhibited a compressive strength of 6.06 MPa, making it the composite scaffold with the highest compressive strength within the scope of this study. Optimal properties for scaffolds include having compressive strengths of 2 to 12 MPa for cancellous bones and 130 to 180 MPa for cortical bones [36]. Therefore, the PCLDMA-60 sample is in the compressive strength range that can be used for cancellous bones.

The blending of polymers is known to be an essentially endothermic process, and thus macroscopically homogeneous-appearing polymer blends result in systems that are microscopically heterogeneous but with an extremely high degree of dispersion. Due to the high viscosity of the polymer mixtures, which prevents macroscopic phase separation, but does not significantly inhibit the mobility of parts of the flexible chain molecules, these microscopically heterogeneous polymer blends are formed. However, it is quite clear that if the two polymers are mutually insoluble, the spontaneous separation of such artificially prepared mixtures into macroscopically distinct phases must proceed at infinitesimal rates because of the enormous viscosity of the system. It has been observed that the mechanical properties of micro-heterogeneous polymer blends depend on the ratio of polymers and exhibit different maximums or minimums in polymer solutions that cannot be obtained in the case of real polymers. Styrene–polybutadiene polymer blends can be shown as the best example of this situation. In cases where the concentration in styrene–polybutadiene polymer mixtures is higher in favor of either of these polymers, the phase separation takes a long time and cannot be seen with the naked eye since the phase separation between the polymers is very slow. However, when these two polymers are blended at a ratio of 50–50%, the organization of the polymer chains in the mixture occurs in a very different way than when the concentration is higher in favor of either of these polymers, and phase separation occurs [37]. In this study, the chemical formulation of the commercial product p-PLA, which is one of the polymers forming the composite structure, is unknown. For this reason, it is thought that the mixtures made with the synthesized PCLDMA polymer may have similar properties to the styrene–polybutadiene polymer mixtures and this may have affected the mechanical properties of the composite scaffolds [14,38,39].

Patricio et al. conducted a study in 2013 where they created PCL/PLA composites with different weight ratios (70/30% and 50/50%) using a physical blending technique known as solvent casting. Upon analysis, it was seen that the composite with a weight ratio of 70/30 PCL/PLA exhibited a significantly smoother surface, with little droplets embedded in the material. In contrast, the composite with a weight ratio of 50/50 PCL/PLA displayed a surface with uniformly distributed material droplets [14].

Also, in their investigation of composite macroporous micro/nanofiber scaffolds composed of polylactic acid (PLA) and polycaprolactone (PCL) in the year 2020, Liu and colleagues discerned that when the PCL content remained below 30%, the compression modulus of the PLA/PCL composite scaffold exhibited a pronounced augmentation correlating with increasing PCL content. Nevertheless, when the PCL content surpassed 45% (specifically, at ratios of 45% and 50%), they observed a conspicuous lack of miscibility between the two polymers, leading to evident phase separation [11]. The results indicate a mutual reinforcement of the findings of Patricio et al. [14] and Liu et al. [11].

### 3.3. In Vitro Degradation of PCLDMA/p-PLA Composite Scaffolds

**Figure 8:**
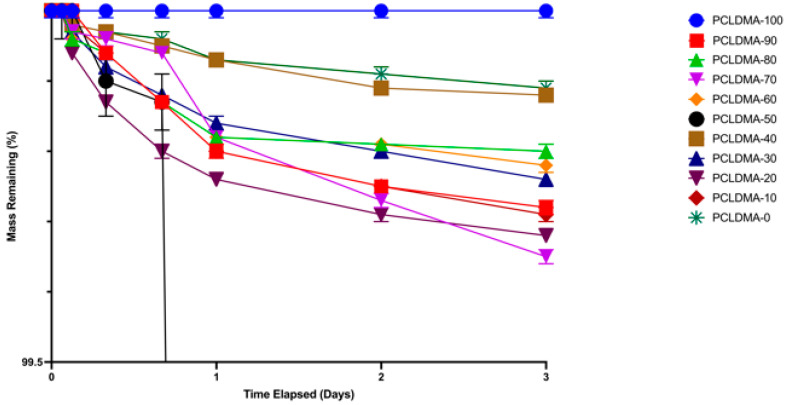
Weight loss rate of PCLDMA/P-PLA composite 3D-printed scaffolds with various contents of PCLDMA (data are given as mean ± SD (*n* = 3)).

In bone tissue engineering applications, appropriate degradation within the physiological milieu is a fundamental hallmark of scaffold functionality. An assessment of the degradation characteristics of the PCLDMA/p-PLA 3D-printed composite scaffolds was undertaken through the consideration of weight loss percentage. The weight loss of the scaffolds over the 3-day period was depicted in Figure 8. As observed in the graph, the weight loss of the scaffolds during this process occurred at a notably gradual pace, with a rating ranging from 0% to 0.4%. These findings align with the degradation rate investigations conducted by Lam et al. on 80,000 molecular-weight PCL polymers [29], as well as the degradation studies carried out by Liu et al. on PCL/PLA composite microfiber scaffolds [11].

### 3.4. Surface Roughness and Porosity of Scaffolds

**Figure 9:**
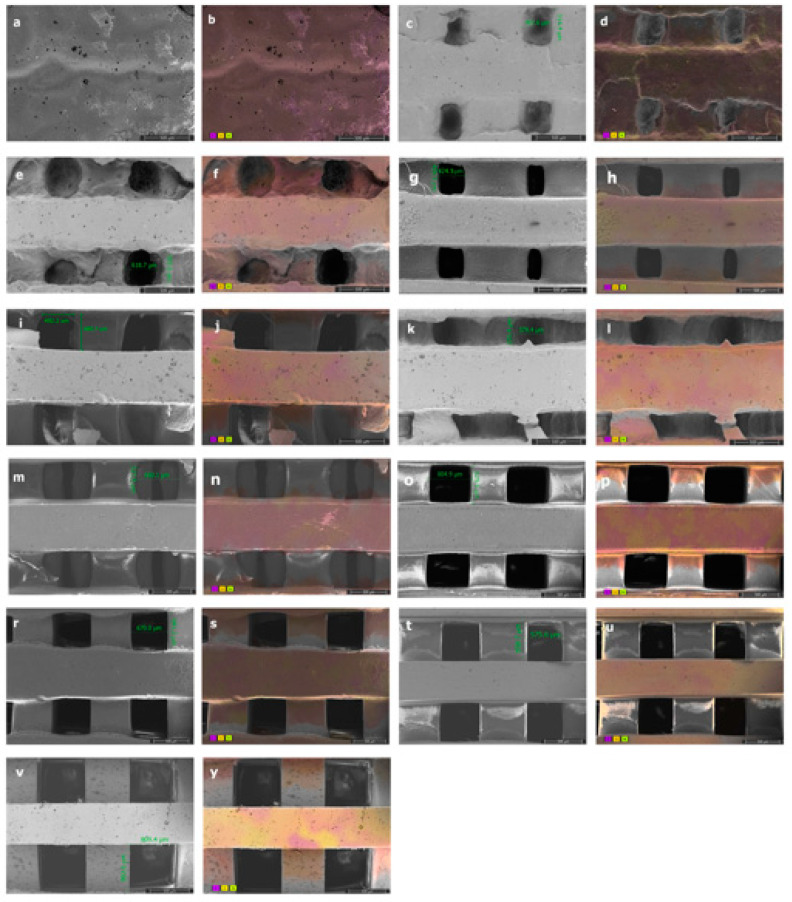
SEM images with pore sizes of (**a**) PCLDMA-100, (**c**) PCLDMA-90, (**e**) PCLDMA-80, (**g**) PCLDMA-70, (**i**) PCLDMA-60, (**k**) PCLDMA-50, (**m**) PCLDMA-40, (**o**) PCLDMA-30, (**r**) PCLDMA-20, (**t**) PCLDMA-10, and (**v**) PCLDMA-0. Color-SEM images with element distributions of (**b**) PCLDMA-100, (**d**) PCLDMA-90, (**f**) PCLDMA-80, (**h**) PCLDMA-70, (**j**) PCLDMA-60, (**l**) PCLDMA-50, (**n**) PCLDMA-40, (**p**) PCLDMA-30, (**s**) PCLDMA-20, (**u**) PCLDMA-10, and (**y**) PCLDMA-0 (C: purple, O: yellow, N: green). Scale bars represent 500 µm.

The characterization parameters of the PCLDMA/P-PLA composite, 3D-printed scaffolds, such as morphology and surface roughness, were observed via SEM, and the element distribution and homogeneity of the polymer mixture were assessed via Color-SEM (Figure 9).

As observed in the SEM images, the increase in the amount of PCLDMA polymer within the 3D composite bone tissue engineering scaffolds resulted in reduced shape accuracy of the 3D structure, a decrease in pore size, and pore blockages. This phenomenon can be attributed to the relatively low quantity of methacrylate side groups on the elongated polymer chain of PCLDMA, leading to an extended curing time under UV light. Conversely, when the amount of p-PLA polymer in the composite structure increased, the shape accuracy of the 3D composite bone tissue engineering scaffold improved, resembling the structure designed using computer-aided design. However, with a higher p-PLA ratio, the stuts forming the structure were shrunk, resulted in larger pore sizes than desired. The optimal properties of scaffolds include having pore sizes in the range of 300–500 μm [36]. Therefore, the PCLDMA-60 sample is considered to be the sample with the most suitable topography and pore size. Color-SEM images illustrate the elements within the structure and their distribution. Upon close examination of these images, it becomes evident that the polymers within the mixture are uniformly dispersed within the structure, but they do not blend homogeneously with each other. This observation aligns with the results obtained from the compression tests. Particularly in the case of the sample named PCLDMA-50, consisting of 50% PCLDMA and 50% p-PLA polymers, we observe pronounced deformations and fractures within the three-dimensional structure. These are attributed to the microscopic heterogeneous mixture formed by PCLDMA and p-PLA polymers [13,14].

Table 2 shows the porosity rates of PCLDMA/p-PLA 3D-printed composite scaffolds.

The porosity of the scaffold models designed is 50%. As can be seen in the table, the porosity rates of 3D-printed composite bone scaffolds vary between 2% and 50%. As the PCLDMA polymer in the structure of composite bone scaffolds increases, the porosity rate decreases. The optimal properties of scaffolds include having an overall porosity of 30–90% [36]. While the samples named PCLDMA-100, PCLDMA-90, PCLDMA-80, and PCLDMA-70 are below this ratio, the porosity ratios of the other samples are in parallel with the ideal bone scaffold properties. These results are also supported by SEM analysis.

### 3.5. Cell Viability

**Figure 10:**
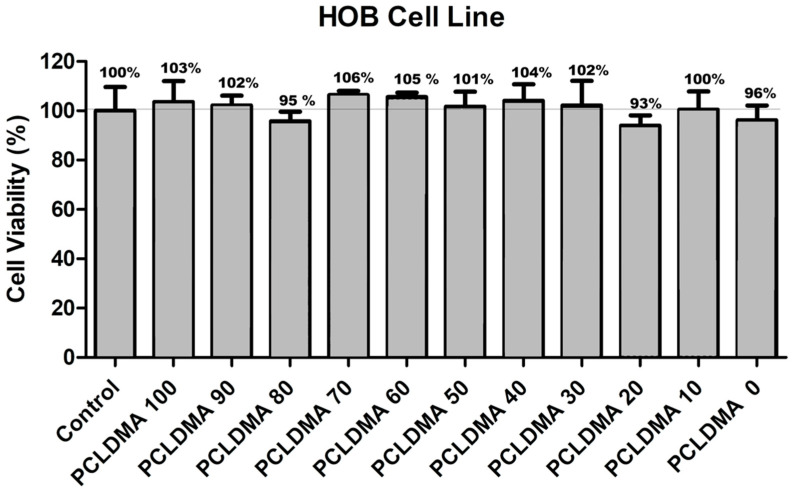
The cell viability results of PCLDMA/p-PLA composite scaffolds with different PCLDMA ratios (*n* = 5). Cell viability assays were performed with HOB cells. As a control, we used DMEM complete media only.

Cell viability assessments were conducted in compliance with the standards outlined in ISO 10993-5, which delineates the procedures for evaluating the in vitro cytotoxicity of medical apparatus. In this investigation, the HOB cell line was utilized as an alternative to the commonly employed L929 cell line stipulated in the conventional testing protocol. The extraction of substances from the bone scaffolds, produced using the SLA 3D printing method, was executed following the protocols specified in ISO 10993-12. This segment of the ISO 10993 directives establishes prerequisites and offers guidance pertaining to the preparation of samples and the selection of reference materials for the assessment of medical devices within biological systems, conforming to one or more sections within the ISO 10993 framework. Three different doses, consisting of 50%, 70%, and 100% purity, were applied to the HOB cell line, and the MTT method was employed to determine cell viability. Figure 10 presents the effects of pure extracts on cell viability. After 72 h, it was observed that the pure extracts did not reduce the viability of the HOB cell line below 90% for any of the samples. Additionally, in some groups, cell viability exceeded 100%.

When the previous studies were examined, it was observed that the results were consistent with the existing literature [25,40,41]. This compatibility can be attributed to several factors. Firstly, the PCL-based PCLDMA polymer and the PLA-based photocurable p-PLA polymer used in the bone scaffolds are FDA-approved biocompatible materials [41]. Secondly, the bone scaffolds obtained from the SLA-type 3D printer underwent thorough washing with appropriate solutions after printing, ensuring the complete removal of any toxic materials. Thirdly, as demonstrated in the degradation test, it was found that they did not degrade in a manner that left behind toxic residues. According to the cell viability tests, PCLDMA/p-PLA composite 3D scaffolds with different PCLDMA ratios showed biocompatibility with HOB living cells.

### 3.6. Fluorescent Microscopy

**Figure 11:**
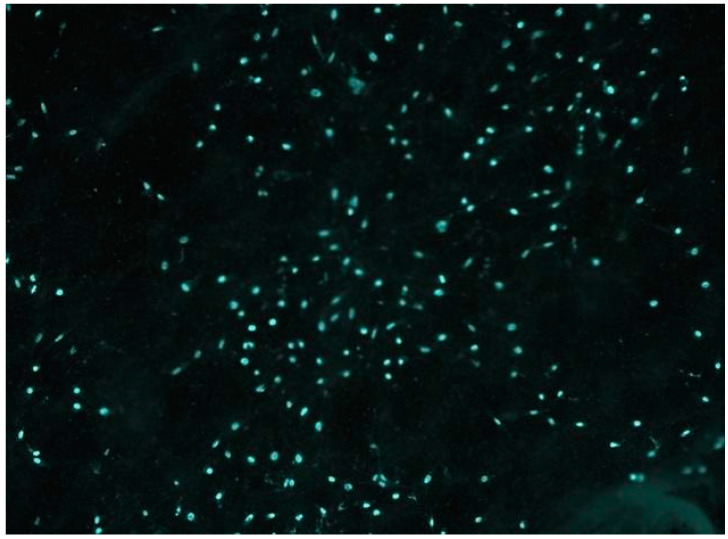
Fluorescence microscope image of the DAPI labelled PCLDMA-60 scaffold with HOB cells. The magnification factor of the microscope in use is set at a twenty-fold increase.

**Figure 12:**
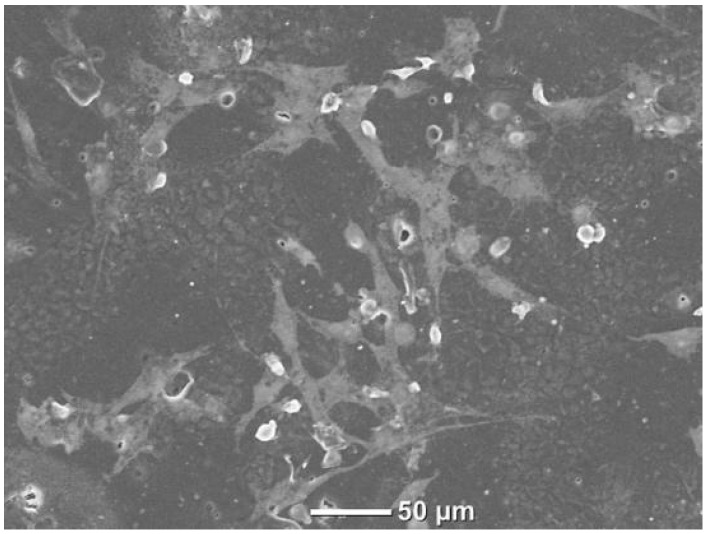
SEM image of PCLDMA-60 scaffold with HOB cells.

Fluorescent microscope images (Figure 11) and SEM images with HOB cells (Figure 12) were taken to obtain more information about cell adhesion on the surface of the sample named PCLDMA-60. The reason for choosing the sample named PCLDMA-60 is that, according to the results of physical, chemical, structural, and cytotoxicity tests, it is considered to have the optimum properties that a bone scaffold should possess.

## 4. Conclusions

This research aimed to synthesize a cationic PCLDMA polymer and assess its suitability for use in bone tissue engineering composite scaffolds. We verified that the polymer was synthesized successfully by employing stringent characterization methods, such as FTIR and NMR analysis. Using SLA 3D printing technology, composite scaffolds were manufactured utilizing different proportions of the synthesized PCLDMA polymer and p-PLA polymer. Compared to individual polymers, the composite scaffolds’ geometry accuracy and mechanical integrity were significantly enhanced. The PCLDMA-60 sample exhibited the most favorable characteristics regarding topography and pore size of all the evaluated compositions. The results of compressive strength experiments exhibited variability in value contingent upon the PCLDMA content, whereas porosity rates spanned a range from 2% to 50%. Degradation studies revealed a gradual increase in degradation rates over three days. Experiments on cell viability validated the biocompatibility of composite scaffolds of varying degrees of purity. The scaffolds exhibited the desired attributes for bone tissue engineering, indicating their promise for subsequent in vivo investigations. Specifically, the PCLDMA-60 composition emerged as a promising candidate for further investigation as a potential substitute for cancellous bone.

## Figures and Tables

**Table 1:** Formulations of Bioresins.

Name	PCLDMA	p-PLA
PCLDMA-100	100%	0%
PCLDMA-90	90%	10%
PCLDMA-80	80%	20%
PCLDMA-70	70%	30%
PCLDMA-60	60%	40%
PCLDMA-50	50%	50%
PCLDMA-40	40%	60%
PCLDMA-30	30%	70%
PCLDMA-20	20%	80%
PCLDMA-10	10%	90%
PCLDMA-0	0%	100%

**Table 2:** Porosity Rates (%) of 3D composite bone tissue engineering scaffolds.

Name	PCLMA (%)	P-PLA (%)	Porosity (%)
PCLDMA-100	100	0	2 (±0.050 SD)
PCLDMA-90	90	10	8 (±0.040 SD)
PCLDMA-80	80	20	12 (±0.025 SD)
PCLDMA-70	70	30	20 (±0.030 SD)
PCLDMA-60	60	40	40 (±0.030 SD)
PCLDMA-50	50	50	Invalid
PCLDMA-40	40	60	45 (±0.027 SD)
PCLDMA-30	30	70	50 (±0.022 SD)
PCLDMA-20	20	80	50 (±0.045 SD)
PCLDMA-10	10	90	50 (±0.038 SD)
PCLDMA-0	0	100	50 (±0.029 SD)

## Data Availability

The data that have been used are confidential.
